# Crystal structure and Hirshfeld surface analysis of ethyl 2-(7-chloro-3-methyl-2-oxo-1,2-di­hydro­quinoxalin-1-yl)acetate

**DOI:** 10.1107/S2056989024002664

**Published:** 2024-03-26

**Authors:** Nour El Hoda Mustaphi, Fatima Ezzahra Aboutofil, Lamyae El Houssni, Eiad Saif, Joel T. Mague, Karim Chkirate, El Mokhtar Essassi

**Affiliations:** a Laboratory of Heterocyclic Organic Chemistry URAC 21, Pharmacochemistry Competence Center, Av. Ibn Battouta, BP 1014, Faculty of Sciences, Mohammed V University in Rabat, Morocco; b Ecole Nationale Supérieure de Chimie, Université Ibn TofaÏl, Kénitra, Morocco; c Department of Computer and Electronic Engineering Technology, Sanaa Community College, Sanaa, Yemen; d Department of Chemistry, Tulane University, New Orleans, LA 70118, USA; Katholieke Universiteit Leuven, Belgium

**Keywords:** crystal structure, C—H⋯π(ring) inter­action, π-stacking, hydrogen bond, quinoxaline

## Abstract

The quinoxaline moiety in the title mol­ecule, C_13_H_13_ClN_2_O_3_, is almost planar (r.m.s. deviation of the fitted atoms = 0.033 Å). In the crystal, C—H⋯O hydrogen bonds plus slipped π-stacking and C—H⋯π(ring) inter­actions generate chains of mol­ecules extending along the *b*-axis direction. The chains are connected by additional C—H⋯O hydrogen bonds. Hirshfeld surface analysis indicates that the most important contributions to the crystal packing are from H⋯H (37.6%), H⋯O/O⋯H (22.7%) and H⋯Cl/Cl⋯H (13.1%) inter­actions.

## Abstract 2

The quinoxaline moiety is almost planar and the dihedral angle between the mean planes through the two constituent six-membered rings is 2.1 (2)°. In the crystal, C—H⋯O hydrogen bonds together with slipped π-stacking and C—H⋯π(ring) inter­actions generate chains of mol­ecules extending along the *b*-axis direction. The chains are connected by additional C—H⋯O hydrogen bonds.

## 1. Chemical context

Nitro­gen-based structures have attracted more attention in recent years because of their inter­esting properties in structural and inorganic chemistry (Faraj *et al.*, 2022 ▸; Chkirate *et al.*, 2022*a* ▸,*b* ▸, 2023 ▸; Al Ati *et al.*, 2024 ▸). The family of quinoxalines, particularly those containing the 2-oxoquinoxaline moiety, is important in medicinal chemistry because of their wide range of pharmacological applications such as anti­bacterial activity (Chkirate *et al.*, 2022*c* ▸) and as potential anti­cancer agents (Abad *et al.*, 2023 ▸). In particular, 3-methyl-2-oxoquinoxaline is a cytotoxic (Missioui *et al.*, 2022*a* ▸) and anti­convulsant agent (Ibrahim *et al.*, 2013 ▸) and has anti-COVID-19 and anti-Alzheimer’s disease (Missioui *et al.*, 2022*b* ▸) activities. Given the wide range of therapeutic applications for such compounds, and in a continuation of the work already carried out on the synthesis of compounds from 2-oxoquinoxaline, a similar approach gave the title compound, ethyl 2-(7-chloro-3-methyl-2-oxoquinoxaline-1(2*H*)-yl)acetate C_13_H_13_ClN_2_O_3_ (I). Besides the synthesis, we also report the mol­ecular and crystalline structures along with a Hirshfeld surface analysis. 
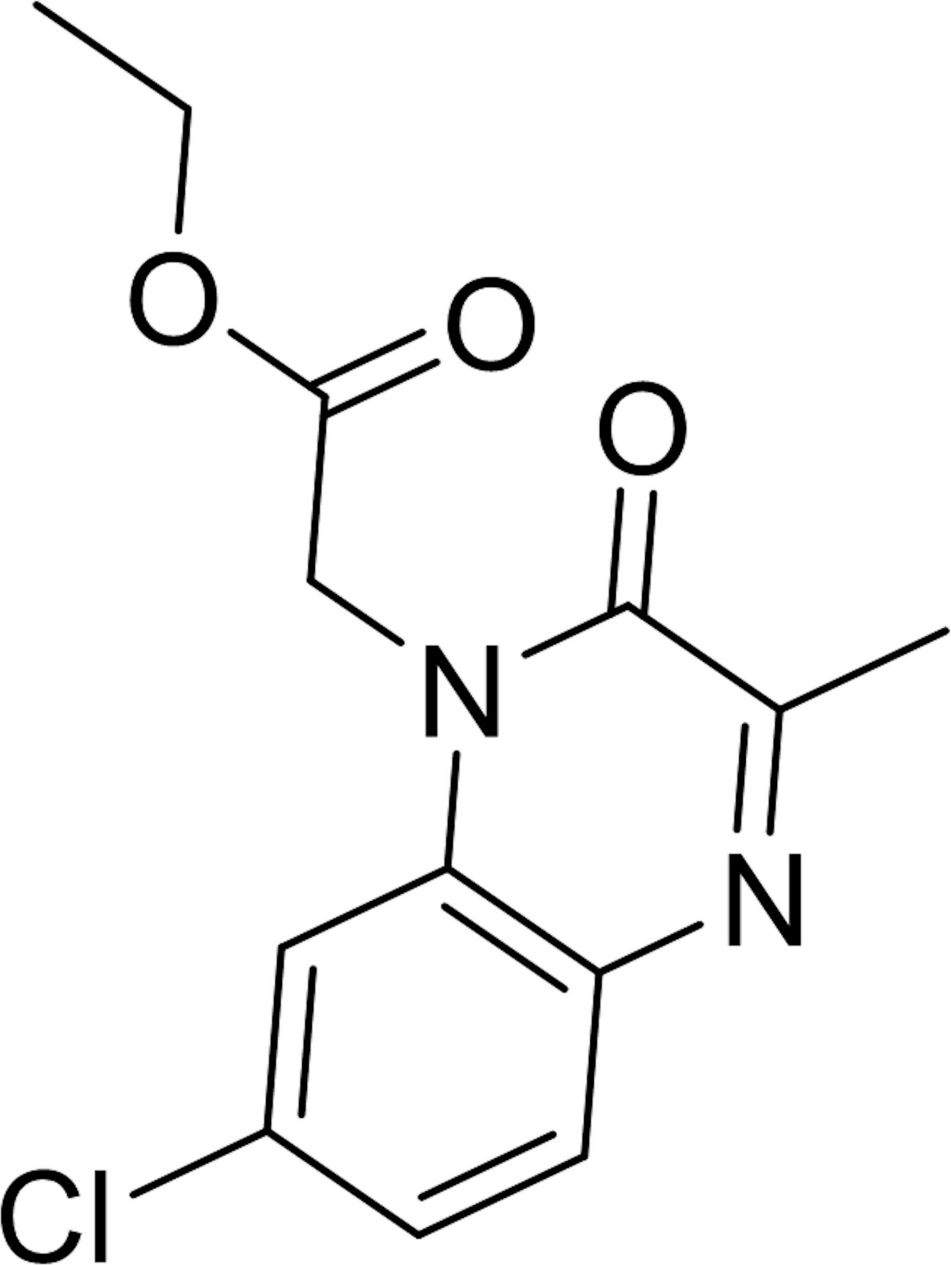


## 2. Structural commentary

**Figure 1:**
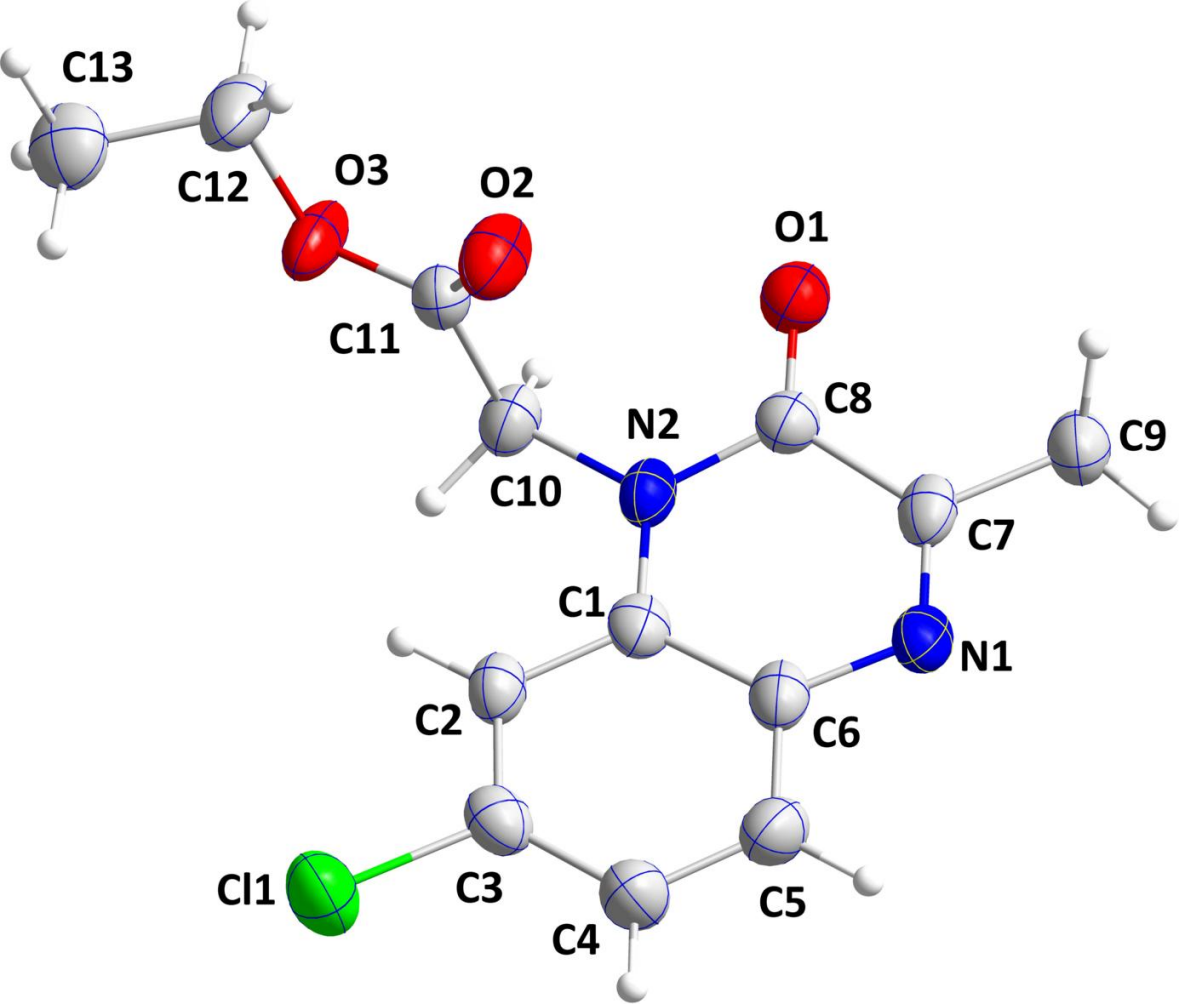
The title mol­ecule with labeling scheme and 50% probability ellipsoids.

The quinoxaline moiety is almost planar (r.m.s. deviation of the fitted atoms = 0.033 Å) with largest deviations being observed for atom C8 [0.072 (5) Å] to one side and atom N2 [−0.072 (5) Å] on the other side of the mean plane. The dihedral angle between the mean planes of the two six-membered rings making up the quinoxaline moiety is 2.1 (2)°. The ester group is rotated well out of the plane of the quinoxaline moiety, as indicated by the C8—N2—C10—C11 torsion angle of −88.2 (5)° (Fig. 1 ▸).

## 3. Supra­molecular features

**Figure 2:**
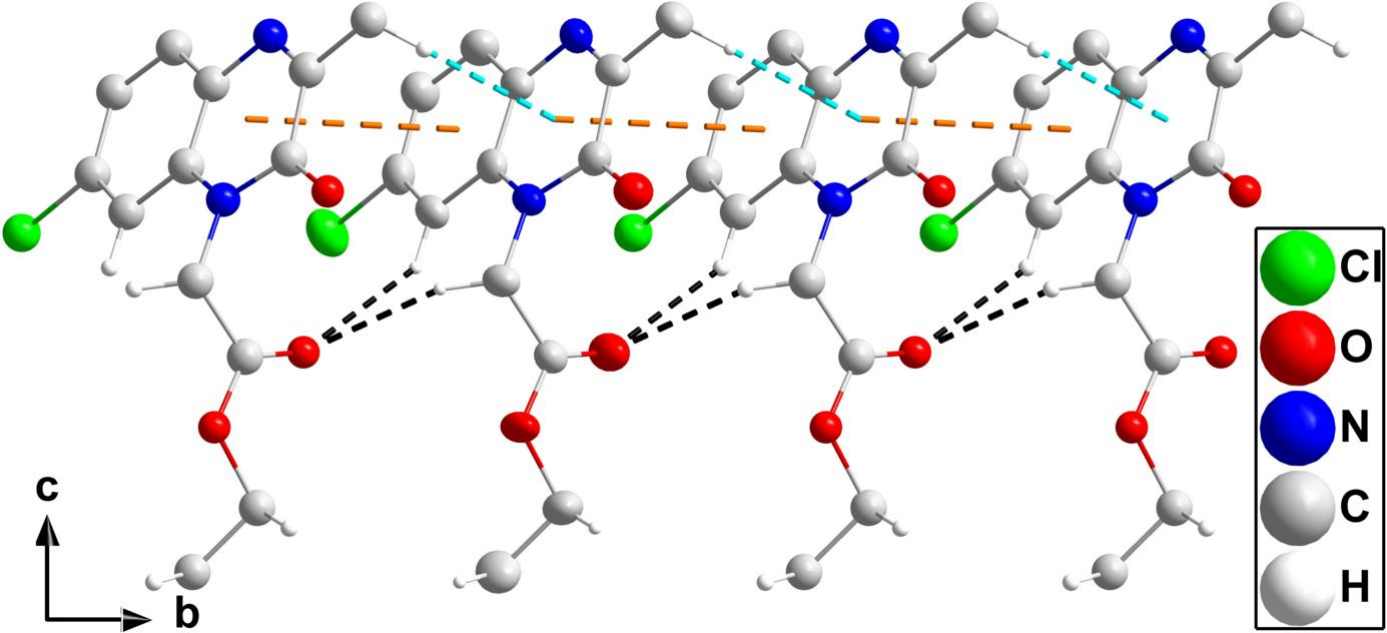
A portion of one chain viewed along the *a*-axis direction with C—H⋯O hydrogen bonds and C—H⋯π(ring) inter­actions depicted, respectively, by black and light blue dashed lines. Slipped π-stacking inter­actions are depicted by orange dashed lines and non-inter­acting hydrogen atoms are omitted for clarity.

**Figure 3:**
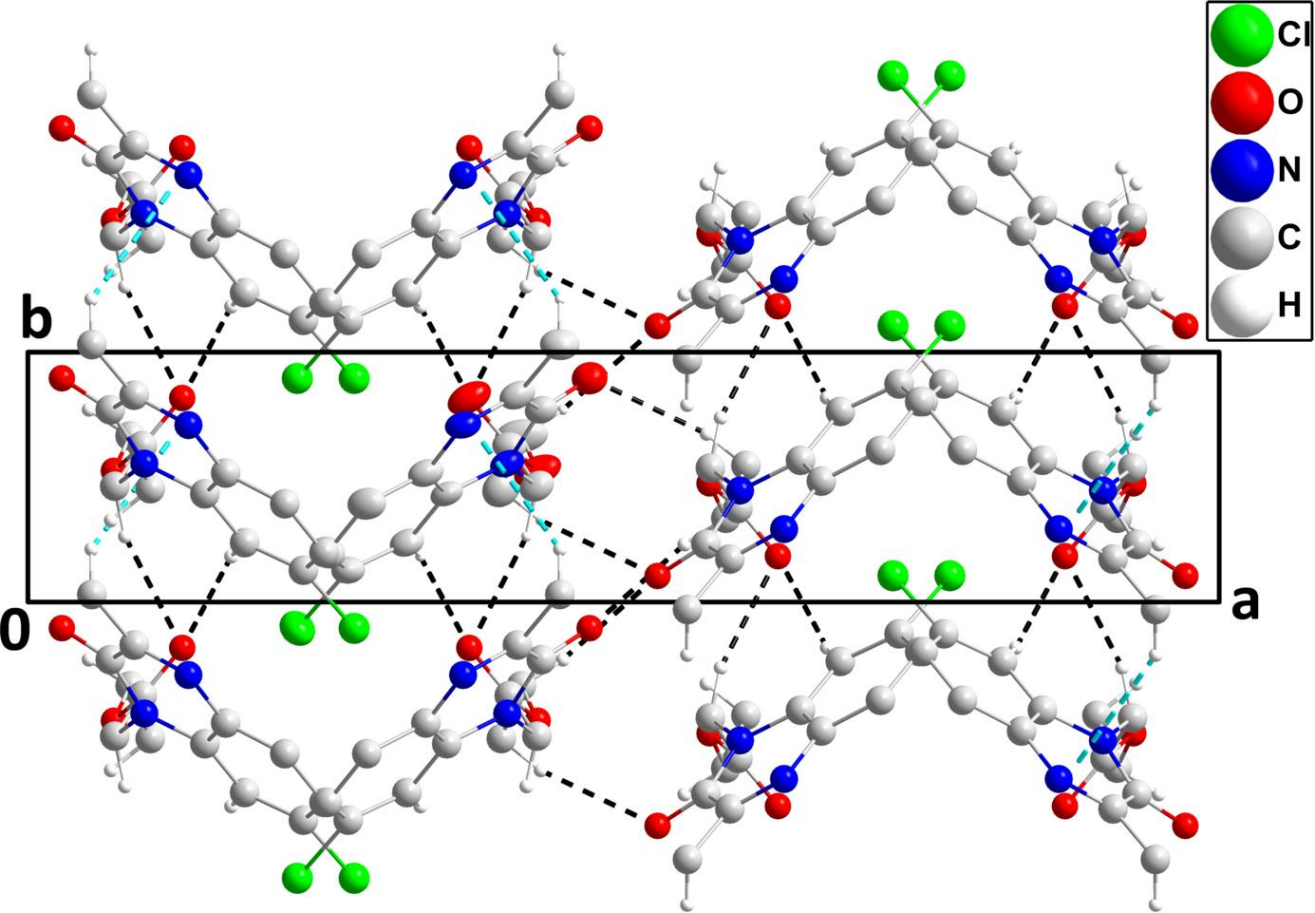
Packing viewed along the *c*-axis direction with C—H⋯O hydrogen bonds and C—H⋯π(ring) inter­actions depicted, respectively, by black and light-blue dashed lines. Non-inter­acting hydrogen atoms and π-stacking inter­actions are omitted for clarity.

In the crystal, C2—H2⋯O2 and C10—H10*A*⋯O2 hydrogen bonds reinforced by C9—H9*A*⋯*Cg*1 inter­actions (Table 1 ▸) and slipped π-stacking inter­actions between the C1/C6/N1/C7/C8/N2 and C1–C6 rings [centroid–centroid distance = 3.756 (3) Å, dihedral angle = 2.1 (2)°, slippage = 1.39 Å] lead to the formation of chains of mol­ecules extending along the *b*-axis direction (Fig. 2 ▸). The chains are connected by C12—H12*A*⋯O1 and C13—H13*A*⋯O1 hydrogen bonds (Table 1 ▸), which form the full three-dimensional structure (Fig. 3 ▸).

## 4. Hirshfeld surface analysis

**Figure 4:**
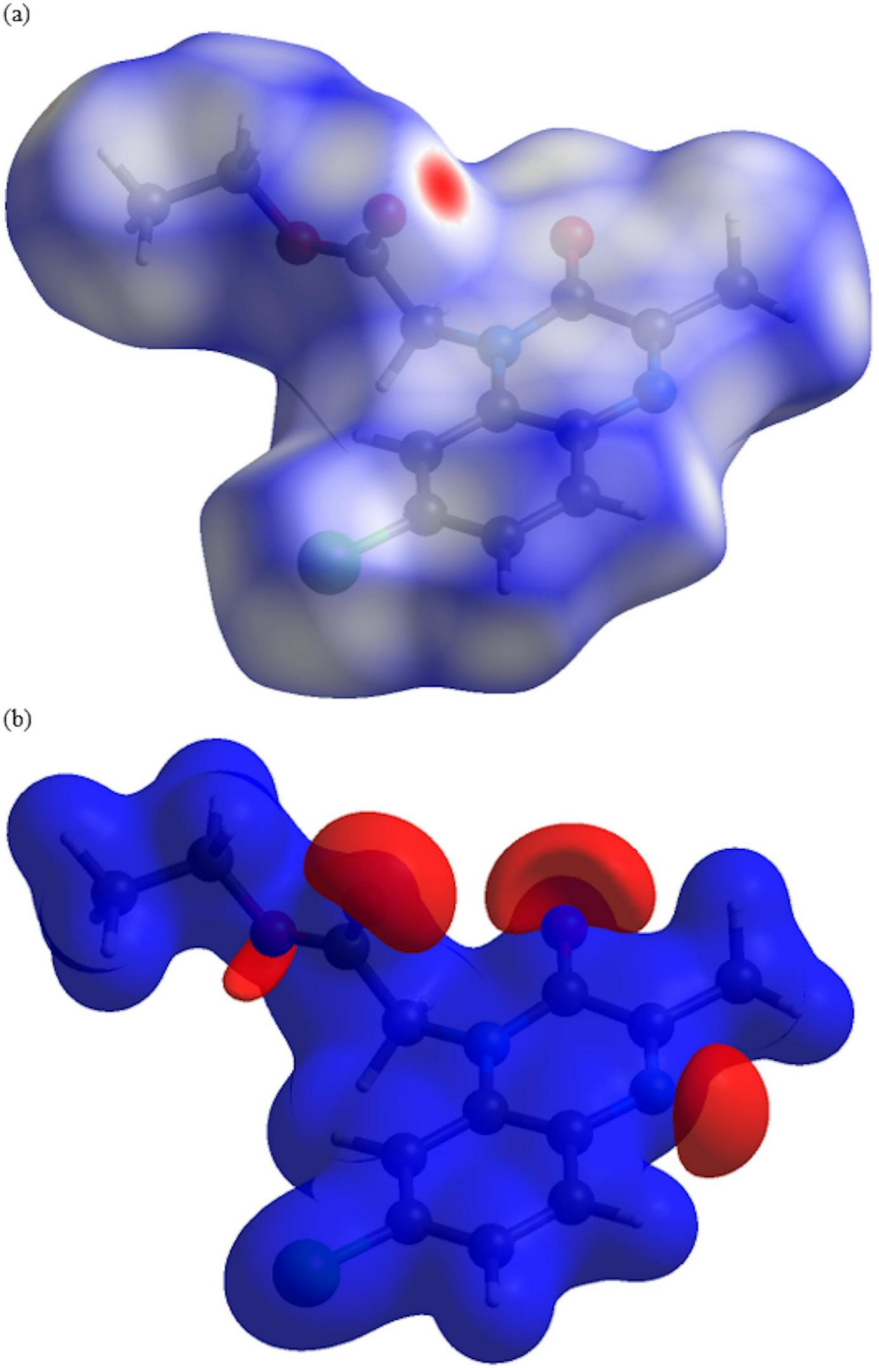
(*a*) View of the three-dimensional Hirshfeld surface of the title compound, plotted over *d*_norm_ and (*b*) view of the three-dimensional Hirshfeld surface of the title compound plotted over electrostatic potential energy using the STO-3 G basis set at the Hartree–Fock level of theory.

**Figure 5:**
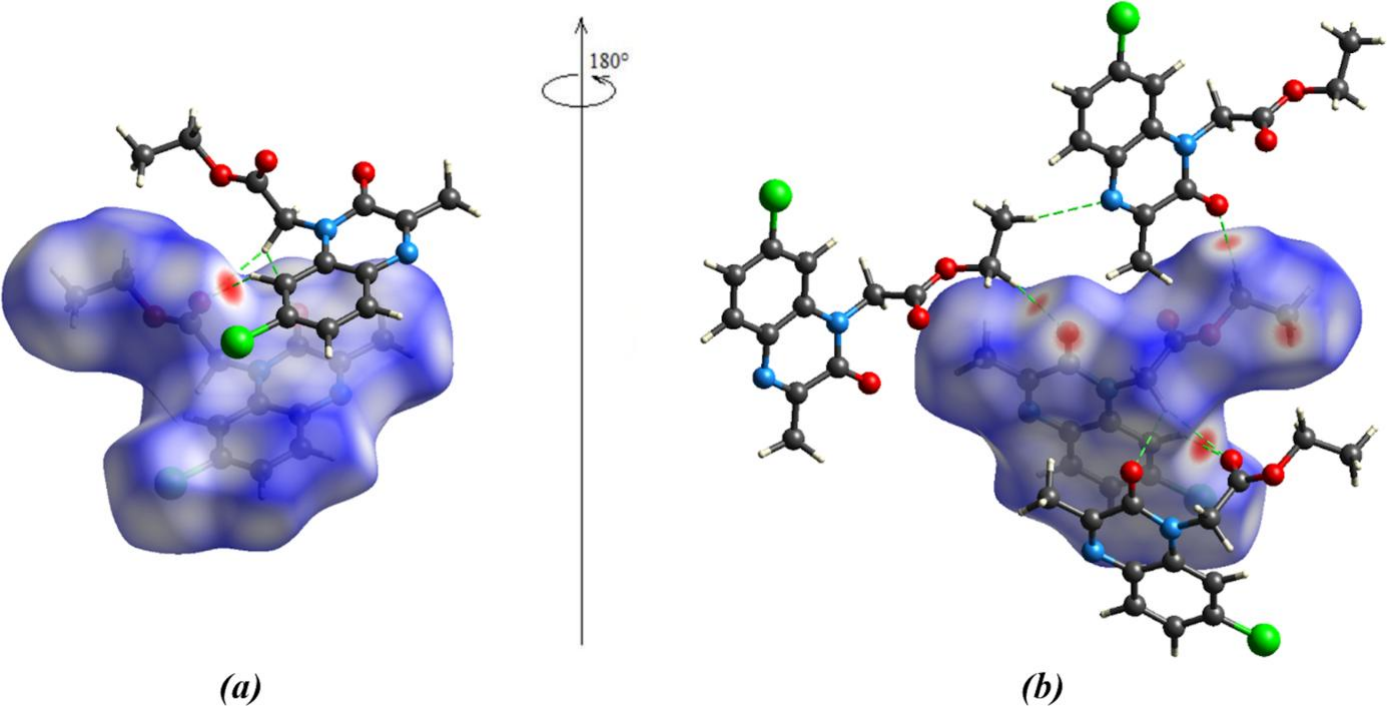
(*a*) Front and (*b*) back sides of the three-dimensional Hirshfeld surface of the compound mapped over *d*_norm_.

*CrystalExplorer* (Turner *et al.*, 2017 ▸) was used to investigate and visualize the inter­molecular inter­actions of (I). The Hirshfeld surface plotted over *d*_norm_ in the range −0.2466 to 1.0065 a.u. is shown in Fig. 4 ▸ *a*. The electrostatic potential using the STO-3G basis set at the Hartree–Fock level of theory and mapped on the Hirshfeld surface over the range ±0.05 a.u. clearly shows the positions of close inter­molecular contacts in the compound (Fig. 4 ▸ *b*). The positive electrostatic potential (blue region) over the surface indicates hydrogen-donor potential, whereas the hydrogen-bond acceptors are represented by negative electrostatic potential (red region). In the standard *d*_norm_ surface (Fig. 5 ▸), the C—H⋯O hydrogen bonds to the closest neighboring mol­ecules are depicted by green dashed lines.

**Figure 6:**
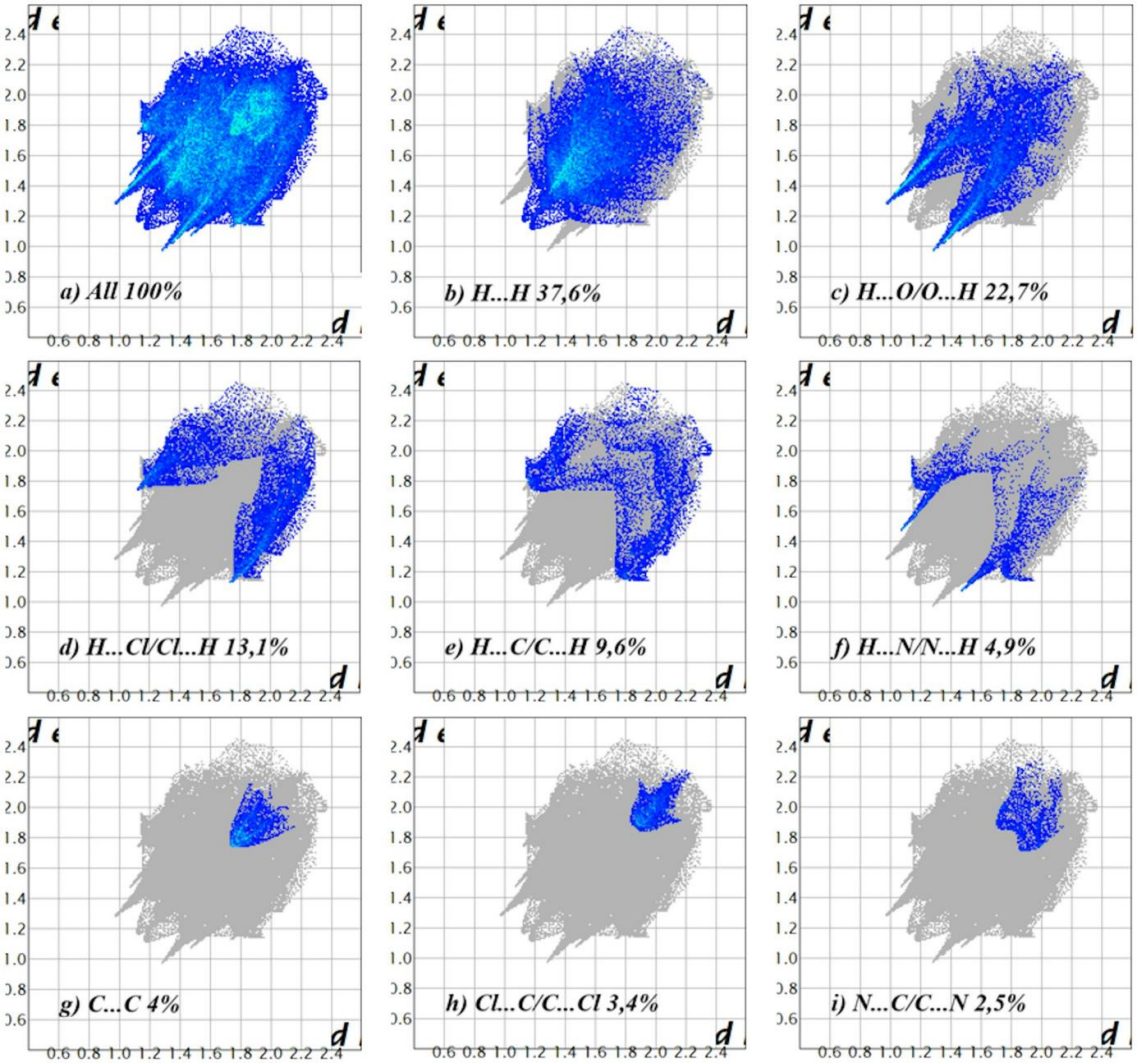
The full two-dimensional fingerprint plots for the title compound, showing (*a*) all inter­actions, and delineated into (*b*) H⋯H, (*c*) H⋯O/O⋯H, (*d*) H⋯Cl/Cl⋯H, (*e*) H⋯C/C⋯H, (*f*) H⋯N/N⋯H, (*g*) C⋯C, (*h*) Cl⋯C/C⋯Cl and (i) N⋯C/C⋯N inter­actions. The *d_i_* and *d_e_* values are the closest inter­nal and external distances (in Å) from given points on the Hirshfeld surface.

The overall two-dimensional fingerprint plot (McKinnon *et al.*, 2007 ▸) is shown in Fig. 6 ▸ *a*, while those delineated into H⋯H, H⋯O/O⋯H, H⋯Cl/Cl⋯H, H⋯C/C⋯H, H⋯N/N⋯H, C⋯C, Cl⋯C/C⋯Cl and N⋯C/C⋯N contacts are illustrated in Fig. 6 ▸ *b*–*i*, respectively, together with their relative contributions to the Hirshfeld surface (HS). The most important inter­action is H⋯H, contributing 37.6% to the overall crystal packing, which is reflected in Fig. 6 ▸ *b* as widely scattered points of high density due to the large hydrogen content of the mol­ecule, with the tip at *d*_e_ = *d*_i_ = 1.16 Å. The H⋯O/O⋯H inter­actions shown by the pair of characteristic wings in the fingerprint plot delineated into these contacts (22.7% contribution to the HS), Fig. 6 ▸ *c*, has the tips at *d*_e_ + *d*_i_ = 2.25 Å. The pair of scattered points of spikes in the fingerprint plot delineated into H⋯Cl/Cl⋯H, Fig. 6 ▸ *d* (13.1%), have the tips at *d*_e_ + *d*_i_ = 2.84 Å. The H⋯C/C⋯H contacts, Fig. 6 ▸ *e* (9.6%), have the tips at *d*_e_ + *d*_i_ = 2.94 Å. The H⋯N/N⋯H contacts, Fig. 6 ▸ *f*, contribute 4.9% to the HS and appear as a pair of scattered points of spikes with the tips at *d*_e_ + *d*_i_ = 2.53 Å. The C⋯C contacts, Fig. 6 ▸ *g* (4%), have the tips at *d*_e_ + *d*_i_ = 3.46 Å. Finally, the Cl⋯C/C⋯Cl and N⋯C/C⋯N contacts, Fig. 6 ▸ *h*–*i*, contribute only 3.4% and 2.5%, respectively, to the HS and have a low-density distribution of points.

## 5. Database survey

**Figure 7:**
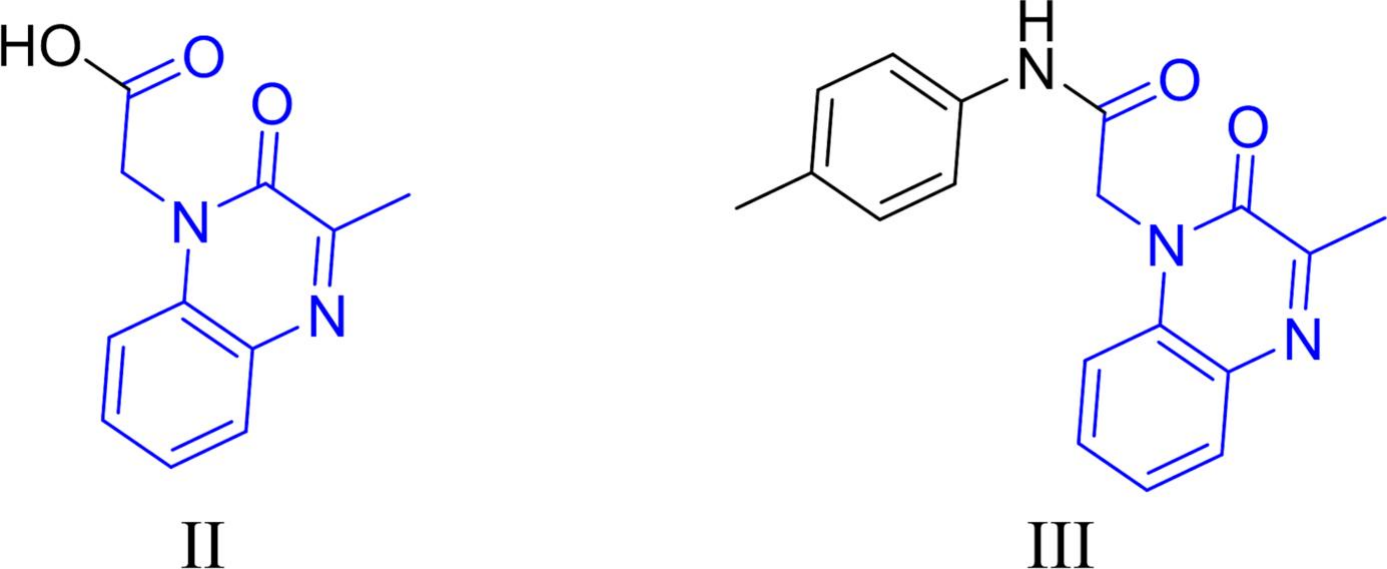
Structures similar to (I): (II) (CSD refcode DEZJAW) and (III) (CSD refcode UGAMEY) obtained during the database search. The search fragment is indicated in blue.

A search of the Cambridge Structural Database (CSD version 5.42, updated May 2021; Groom *et al.*, 2016 ▸) with the 2-(3-methyl-2-oxoquinoxalin-1(2*H*)-yl)acetyl fragment yielded multiple matches. Of these, two had a substituent on C11 comparable to (I) (Fig. 7 ▸). The first compound (II) (refcode DEZJAW; Missioui *et al.*, 2018 ▸) carries a hydroxyl group on C11, while the second one (III) (refcode UGAMEY; Missioui *et al.*, 2023 ▸) carries a *p*-tolyl­azane substituent. The acetic acid part in DEZJAW forms a dihedral angle of −93.62 (11)° with 3-methyl-2-oxoquinoxaline unit. In UGAMEY, the dihedral angles between the mean planes of the *N*-(*p*-tol­yl)acetyl­amide (two positions with occupancies 0.50:0.50) and 3-methyl-2-oxoquinoxaline rings are 104.1 (2) and −71.0 (2)°. As previously mentioned, the ethyl acetate group in (I) is also almost perpendicular to the 3-methyl-2-oxoquinoxaline unit [dihedral angle of −88.2 (5)°], which is approximately the same as in DEZJAW, and in between the two values in UGAMEY.

## 6. Synthesis and crystallization

1.00 g (6.24 mmol) of 7-chloro-3-methyl­quinoxalin-2(1*H*)-one was dissolved in 25 mL of di­methyl­formamide and 1.15 g (6.24 mmol) of ethyl 2-chloro­acetate were added, followed by 1.0 g (7.5 mmol) of potassium bicarbonate, and a spatula tip of BTBA (benzyl­tri­butyl­ammonium chloride) was used as a phase-transfer catalyst. The reaction was stirred for 2 h under reflux at 353 K. When the starting reagents had completely reacted, 500 mL of distilled water were added and a few minutes later the product precipitated. This was filtered off, dried and recrystallized from hot ethanol solution to yield light-yellow plate-like crystals of the title compound. ^1^H NMR (300 MHz, CDCl_3_) δ ppm: 1.21 (*t*, 3H, CH_3_, *J* = 6 Hz); 2.07 (*s*, 3H, CH_3_); 4.16 (*quin*, 2H, CH_2_); 4.59 (*s*, 2H, CH_2_); 7.18–7.87 (*m*, 3H, CH_arom_). ^13^C NMR (75 MHz, CDCl_3_) δ ppm: 14.1 (CH_3_); 21.3 (CH_3_); 51.6(CH_2_); 61.0 (CH_2_); 123.3–125.7 (CH_arom_); 131.2–155.6 (C_q_); 155.7 (C=O); 167.6 (C=O).

## 7. Refinement

Crystal data, data collection and structure refinement details are summarized in Table 2 ▸. The structure was refined as an inversion twin. Hydrogen atoms were were included as riding contributions in idealized positions and refined isotropically.

## Supplementary Material

Crystal structure: contains datablock(s) global, I. DOI: 10.1107/S2056989024002664/vm2299sup1.cif

Structure factors: contains datablock(s) I. DOI: 10.1107/S2056989024002664/vm2299Isup2.hkl

Supporting information file. DOI: 10.1107/S2056989024002664/vm2299Isup3.cml

CCDC reference: 2342203

Additional supporting information:  crystallographic information; 3D view; checkCIF report

## Figures and Tables

**Table 1:** Hydrogen-bond geometry (Å, °) *Cg*1 is the centroid of the C1/C6/N1/C7/C8/N2 ring.

*D*—H⋯*A*	*D*—H	H⋯*A*	*D*⋯*A*	*D*—H⋯*A*
C2—H2⋯O2^i^	0.95	2.39	3.211 (6)	145
C9—H9*A*⋯*Cg*1^ii^	0.98	2.73	3.591 (6)	147
C10—H10*A*⋯O2^i^	0.99	2.59	3.535 (7)	159
C12—H12*A*⋯O1^iii^	0.99	2.49	3.471 (9)	170
C13—H13*A*⋯O1^iv^	0.98	2.49	3.427 (7)	160

Symmetry codes: (i) 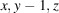 ; (ii) 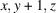 ; (iii) 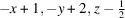 ; (iv) 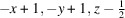 .

**Table 2:** Experimental details

Crystal data	
Chemical formula	C_13_H_13_ClN_2_O_3_
*M*_r_	280.70
Crystal system, space group	Orthorhombic, *P* *c* *a*2_1_
Temperature (K)	150
*a*, *b*, *c* (Å)	22.8042 (11), 4.7826 (2), 11.7421 (6)
*V* (Å^3^)	1280.63 (10)
*Z*	4
Radiation type	Cu *K*α
μ (mm^−1^)	2.71
Crystal size (mm)	0.21 × 0.14 × 0.13

Data collection	
Diffractometer	Bruker D8 VENTURE PHOTON 3 CPAD
Absorption correction	Multi-scan (*SADABS*; Krause *et al.*, 2015 ▸)
*T*_min_, *T*_max_	0.60, 0.72
No. of measured, independent and observed [*I* > 2σ(*I*)] reflections	22953, 2495, 2468
*R*_int_	0.050
(sin θ/λ)_max_ (Å^−1^)	0.619

Refinement	
*R*[*F* ^2^ > 2σ(*F* ^2^)], *wR*(*F* ^2^), *S*	0.058, 0.160, 1.09
No. of reflections	2495
No. of parameters	175
No. of restraints	1
H-atom treatment	H-atom parameters constrained
Δρ_max_, Δρ_min_ (e Å^−3^)	1.27, −0.32
Absolute structure	Refined as an inversion twin
Absolute structure parameter	0.17 (4)

Computer programs: *APEX4* and *SAINT* (Bruker, 2021 ▸), *SHELXS* and *SHELXTL* (Sheldrick, 2008 ▸), *SHELXL2018/1* (Sheldrick, 2015 ▸) and *DIAMOND* (Brandenburg & Putz, 2012 ▸).

## supplementary crystallographic information

### Crystal data

**Table:**

C_13_H_13_ClN_2_O_3_	*D*_x_ = 1.456 Mg m^−^^3^
*M**_r_* = 280.70	Cu *K*α radiation, λ = 1.54178 Å
Orthorhombic, *P**c**a*2_1_	Cell parameters from 9927 reflections
*a* = 22.8042 (11) Å	θ = 7.8–72.1°
*b* = 4.7826 (2) Å	µ = 2.71 mm^−^^1^
*c* = 11.7421 (6) Å	*T* = 150 K
*V* = 1280.63 (10) Å^3^	Column, colourless
*Z* = 4	0.21 × 0.14 × 0.13 mm
*F*(000) = 584	

### Data collection

**Table:**

Bruker D8 VENTURE PHOTON 3 CPAD diffractometer	2495 independent reflections
Radiation source: INCOATEC IµS micro–focus source	2468 reflections with *I* > 2σ(*I*)
Mirror monochromator	*R*_int_ = 0.050
Detector resolution: 7.3910 pixels mm^-1^	θ_max_ = 72.6°, θ_min_ = 3.9°
φ and ω scans	*h* = −28→28
Absorption correction: multi-scan (*SADABS*; Krause *et al.*, 2015)	*k* = −5→5
*T*_min_ = 0.60, *T*_max_ = 0.72	*l* = −14→14
22953 measured reflections	

### Refinement

**Table:**

Refinement on *F*^2^	Secondary atom site location: difference Fourier map
Least-squares matrix: full	Hydrogen site location: inferred from neighbouring sites
*R*[*F*^2^ > 2σ(*F*^2^)] = 0.058	H-atom parameters constrained
*wR*(*F*^2^) = 0.160	*w* = 1/[σ^2^(*F*_o_^2^) + (0.0982*P*)^2^ + 1.0105*P*] where *P* = (*F*_o_^2^ + 2*F*_c_^2^)/3
*S* = 1.09	(Δ/σ)_max_ < 0.001
2495 reflections	Δρ_max_ = 1.27 e Å^−^^3^
175 parameters	Δρ_min_ = −0.32 e Å^−^^3^
1 restraint	Absolute structure: Refined as an inversion twin
Primary atom site location: dual	Absolute structure parameter: 0.17 (4)

### Special details

**Table:**

Experimental. The diffraction data were obtained from 16 sets of frames, each of width 0.5° in ω or φ, collected with scan parameters determined by the "strategy" routine in *APEX4*. The scan time was θ-dependent and ranged from 5 to 15 sec/frame.
Geometry. All esds (except the esd in the dihedral angle between two l.s. planes) are estimated using the full covariance matrix. The cell esds are taken into account individually in the estimation of esds in distances, angles and torsion angles; correlations between esds in cell parameters are only used when they are defined by crystal symmetry. An approximate (isotropic) treatment of cell esds is used for estimating esds involving l.s. planes.
Refinement. Refinement of F^2^ against ALL reflections. The weighted R-factor wR and goodness of fit S are based on F^2^, conventional R-factors R are based on F, with F set to zero for negative F^2^. The threshold expression of F^2^ > 2sigma(F^2^) is used only for calculating R-factors(gt) etc. and is not relevant to the choice of reflections for refinement. R-factors based on F^2^ are statistically about twice as large as those based on F, and R- factors based on ALL data will be even larger. H-atoms attached to carbon were placed in calculated positions (C—H = 0.95 - 0.98 Å). All were included as riding contributions with isotropic displacement parameters 1.2 - 1.5 times those of the attached atoms. Refined as a 2-component inversion twin.

### Fractional atomic coordinates and isotropic or equivalent isotropic displacement parameters (Å^2^)

**Table:**

	*x*	*y*	*z*	*U*_iso_*/*U*_eq_	
Cl1	0.22614 (6)	−0.1052 (3)	0.52846 (14)	0.0525 (4)	
O1	0.47152 (17)	0.8955 (8)	0.5839 (3)	0.0431 (9)	
O2	0.3705 (2)	0.8166 (9)	0.3693 (4)	0.0522 (10)	
O3	0.4279 (2)	0.5252 (9)	0.2694 (3)	0.0510 (10)	
N1	0.3653 (2)	0.7081 (9)	0.7912 (4)	0.0376 (9)	
N2	0.40226 (18)	0.5540 (8)	0.5727 (3)	0.0341 (8)	
C1	0.3515 (2)	0.4330 (10)	0.6175 (4)	0.0328 (9)	
C2	0.3181 (2)	0.2376 (10)	0.5565 (4)	0.0357 (10)	
H2	0.329386	0.182454	0.481938	0.043*	
C3	0.2688 (2)	0.1272 (10)	0.6067 (5)	0.0394 (11)	
C4	0.2514 (3)	0.1981 (12)	0.7161 (5)	0.0442 (11)	
H4	0.217756	0.115043	0.749633	0.053*	
C5	0.2844 (3)	0.3931 (11)	0.7754 (5)	0.0444 (12)	
H5	0.273074	0.444011	0.850445	0.053*	
C6	0.3340 (2)	0.5161 (10)	0.7271 (4)	0.0364 (10)	
C7	0.4108 (2)	0.8277 (10)	0.7453 (4)	0.0365 (10)	
C8	0.4309 (2)	0.7692 (10)	0.6277 (4)	0.0346 (10)	
C9	0.4464 (3)	1.0300 (12)	0.8123 (5)	0.0448 (12)	
H9A	0.446634	1.211426	0.773475	0.067*	
H9B	0.486658	0.960415	0.819087	0.067*	
H9C	0.429315	1.051443	0.888356	0.067*	
C10	0.4272 (2)	0.4636 (11)	0.4646 (4)	0.0370 (10)	
H10A	0.418454	0.262610	0.453586	0.044*	
H10B	0.470335	0.484805	0.467859	0.044*	
C11	0.4041 (2)	0.6255 (10)	0.3634 (4)	0.0352 (10)	
C12	0.4143 (4)	0.6646 (14)	0.1619 (5)	0.0614 (18)	
H12A	0.449004	0.769649	0.134877	0.074*	
H12B	0.381753	0.798883	0.173224	0.074*	
C13	0.3973 (3)	0.4535 (14)	0.0764 (6)	0.0529 (14)	
H13A	0.430353	0.326874	0.062697	0.079*	
H13B	0.386631	0.547199	0.005148	0.079*	
H13C	0.363703	0.346276	0.104623	0.079*	

### Atomic displacement parameters (Å^2^)

**Table:**

	*U*^11^	*U*^22^	*U*^33^	*U*^12^	*U*^13^	*U*^23^
Cl1	0.0496 (7)	0.0478 (7)	0.0602 (8)	−0.0075 (5)	0.0026 (6)	−0.0130 (7)
O1	0.0500 (19)	0.042 (2)	0.0375 (17)	0.0019 (15)	0.0010 (15)	−0.0006 (16)
O2	0.073 (3)	0.045 (2)	0.0387 (19)	0.022 (2)	−0.0015 (18)	−0.0020 (17)
O3	0.086 (3)	0.0414 (19)	0.0255 (17)	0.021 (2)	0.0055 (16)	−0.0018 (15)
N1	0.051 (2)	0.0289 (19)	0.0324 (19)	0.0053 (18)	−0.0003 (17)	−0.0023 (16)
N2	0.045 (2)	0.0297 (18)	0.0272 (18)	0.0082 (16)	−0.0025 (16)	−0.0030 (16)
C1	0.041 (2)	0.025 (2)	0.033 (2)	0.0080 (17)	−0.0022 (18)	0.0004 (17)
C2	0.046 (2)	0.031 (2)	0.031 (2)	0.0069 (19)	−0.0023 (17)	−0.0051 (18)
C3	0.045 (3)	0.028 (2)	0.045 (3)	0.0014 (18)	−0.004 (2)	−0.001 (2)
C4	0.049 (3)	0.040 (3)	0.044 (3)	−0.004 (2)	0.004 (2)	−0.003 (2)
C5	0.058 (3)	0.040 (3)	0.036 (3)	0.002 (2)	0.008 (2)	0.000 (2)
C6	0.049 (3)	0.030 (2)	0.030 (2)	0.007 (2)	−0.0062 (18)	−0.0018 (18)
C7	0.049 (3)	0.032 (2)	0.028 (2)	0.010 (2)	−0.008 (2)	−0.0016 (19)
C8	0.042 (2)	0.029 (2)	0.032 (2)	0.004 (2)	0.0002 (18)	0.0014 (18)
C9	0.061 (3)	0.037 (3)	0.036 (3)	−0.001 (2)	−0.004 (2)	−0.006 (2)
C10	0.045 (2)	0.038 (3)	0.028 (2)	0.008 (2)	−0.0005 (19)	−0.003 (2)
C11	0.045 (2)	0.030 (2)	0.030 (2)	−0.0013 (19)	−0.0002 (19)	−0.0033 (18)
C12	0.109 (6)	0.043 (3)	0.032 (3)	0.008 (3)	0.001 (3)	0.003 (2)
C13	0.056 (3)	0.054 (3)	0.049 (3)	0.008 (3)	−0.012 (3)	0.000 (3)

### Geometric parameters (Å, º)

**Table:**

Cl1—C3	1.739 (5)	C5—C6	1.395 (8)
O1—C8	1.220 (6)	C5—H5	0.9500
O2—C11	1.194 (7)	C7—C8	1.482 (6)
O3—C11	1.320 (6)	C7—C9	1.487 (7)
O3—C12	1.460 (7)	C9—H9A	0.9800
N1—C7	1.302 (7)	C9—H9B	0.9800
N1—C6	1.385 (7)	C9—H9C	0.9800
N2—C8	1.379 (6)	C10—C11	1.514 (7)
N2—C1	1.397 (7)	C10—H10A	0.9900
N2—C10	1.456 (6)	C10—H10B	0.9900
C1—C2	1.402 (7)	C12—C13	1.475 (9)
C1—C6	1.405 (7)	C12—H12A	0.9900
C2—C3	1.375 (7)	C12—H12B	0.9900
C2—H2	0.9500	C13—H13A	0.9800
C3—C4	1.387 (8)	C13—H13B	0.9800
C4—C5	1.386 (8)	C13—H13C	0.9800
C4—H4	0.9500		
			
C11—O3—C12	118.0 (4)	N2—C8—C7	115.5 (4)
C7—N1—C6	118.5 (4)	C7—C9—H9A	109.5
C8—N2—C1	121.7 (4)	C7—C9—H9B	109.5
C8—N2—C10	116.4 (4)	H9A—C9—H9B	109.5
C1—N2—C10	121.9 (4)	C7—C9—H9C	109.5
N2—C1—C2	122.3 (4)	H9A—C9—H9C	109.5
N2—C1—C6	117.6 (4)	H9B—C9—H9C	109.5
C2—C1—C6	120.1 (5)	N2—C10—C11	113.4 (4)
C3—C2—C1	118.8 (4)	N2—C10—H10A	108.9
C3—C2—H2	120.6	C11—C10—H10A	108.9
C1—C2—H2	120.6	N2—C10—H10B	108.9
C2—C3—C4	122.4 (5)	C11—C10—H10B	108.9
C2—C3—Cl1	118.5 (4)	H10A—C10—H10B	107.7
C4—C3—Cl1	119.1 (4)	O2—C11—O3	126.2 (5)
C5—C4—C3	118.4 (5)	O2—C11—C10	124.6 (5)
C5—C4—H4	120.8	O3—C11—C10	109.1 (4)
C3—C4—H4	120.8	O3—C12—C13	109.3 (5)
C4—C5—C6	121.3 (5)	O3—C12—H12A	109.8
C4—C5—H5	119.3	C13—C12—H12A	109.8
C6—C5—H5	119.3	O3—C12—H12B	109.8
N1—C6—C5	118.4 (4)	C13—C12—H12B	109.8
N1—C6—C1	122.6 (5)	H12A—C12—H12B	108.3
C5—C6—C1	118.9 (5)	C12—C13—H13A	109.5
N1—C7—C8	123.3 (4)	C12—C13—H13B	109.5
N1—C7—C9	120.1 (4)	H13A—C13—H13B	109.5
C8—C7—C9	116.6 (5)	C12—C13—H13C	109.5
O1—C8—N2	122.1 (4)	H13A—C13—H13C	109.5
O1—C8—C7	122.3 (5)	H13B—C13—H13C	109.5
			
C8—N2—C1—C2	172.3 (4)	C2—C1—C6—C5	2.5 (7)
C10—N2—C1—C2	−6.5 (7)	C6—N1—C7—C8	0.1 (7)
C8—N2—C1—C6	−7.0 (6)	C6—N1—C7—C9	−178.6 (4)
C10—N2—C1—C6	174.2 (4)	C1—N2—C8—O1	−172.2 (4)
N2—C1—C2—C3	179.8 (4)	C10—N2—C8—O1	6.7 (7)
C6—C1—C2—C3	−0.9 (7)	C1—N2—C8—C7	10.5 (6)
C1—C2—C3—C4	−1.2 (8)	C10—N2—C8—C7	−170.6 (4)
C1—C2—C3—Cl1	177.7 (3)	N1—C7—C8—O1	175.5 (5)
C2—C3—C4—C5	1.7 (8)	C9—C7—C8—O1	−5.8 (7)
Cl1—C3—C4—C5	−177.1 (4)	N1—C7—C8—N2	−7.2 (7)
C3—C4—C5—C6	−0.1 (9)	C9—C7—C8—N2	171.5 (4)
C7—N1—C6—C5	−178.5 (4)	C8—N2—C10—C11	−88.2 (5)
C7—N1—C6—C1	4.0 (7)	C1—N2—C10—C11	90.7 (5)
C4—C5—C6—N1	−179.6 (5)	C12—O3—C11—O2	2.6 (9)
C4—C5—C6—C1	−2.0 (8)	C12—O3—C11—C10	−176.1 (5)
N2—C1—C6—N1	−0.7 (6)	N2—C10—C11—O2	2.8 (7)
C2—C1—C6—N1	180.0 (4)	N2—C10—C11—O3	−178.5 (4)
N2—C1—C6—C5	−178.2 (4)	C11—O3—C12—C13	−131.7 (6)

### Hydrogen-bond geometry (Å, º)

*Cg*1 is the centroid of the C1/C6/N1/C7/C8/N2 ring.

**Table:**

*D*—H···*A*	*D*—H	H···*A*	*D*···*A*	*D*—H···*A*
C2—H2···O2^i^	0.95	2.39	3.211 (6)	145
C9—H9*A*···*Cg*1^ii^	0.98	2.73	3.591 (6)	147
C10—H10*A*···O2^i^	0.99	2.59	3.535 (7)	159
C12—H12*A*···O1^iii^	0.99	2.49	3.471 (9)	170
C13—H13*A*···O1^iv^	0.98	2.49	3.427 (7)	160

Symmetry codes: (i) *x*, *y*−1, *z*; (ii) *x*, *y*+1, *z*; (iii) −*x*+1, −*y*+2, *z*−1/2; (iv) −*x*+1, −*y*+1, *z*−1/2.
